# Prenatal Fine Particulate Matter (PM_2.5_) Exposure and Pregnancy Outcomes—Analysis of Term Pregnancies in Poland

**DOI:** 10.3390/ijerph17165820

**Published:** 2020-08-11

**Authors:** Cezary Wojtyla, Karolina Zielinska, Paulina Wojtyla-Buciora, Grzegorz Panek

**Affiliations:** 1 Department of Oncologic Gynecology and Obstetrics, Centre of Postgraduate Medical Education, 231 Czerniakowska St., 00-416 Warsaw, Poland; karolina.anna.zielinska@gmail.com (K.Z.); gmpanek@wp.pl (G.P.); 2 Faculty of Health Sciences, State University of Applied Sciences, 16 Kaszubska St., 62-800 Kalisz, Poland; paulinawojtyla@gmail.com

**Keywords:** air pollution, pregnancy, PM_2.5_, pregnancy outcomes

## Abstract

Air pollution is currently one of the greatest threats to global health. Polish cities are among the most heavily polluted in Europe. Due to air pollution 43,100 people die prematurely in Poland every year. However, these data do not take into account the health consequences of air pollution for unborn children. Thus, the aim of this study was to evaluate the effects of the fine particulate matter air pollution (less than 2.5 μm in diameter) on pregnancy outcomes. An analysis of pregnant women and their children was made using a questionnaire survey from a nationwide study conducted in 2017. Questionnaires from 1095 pregnant women and data from their medical records were collected. An analysis of air pollution in Poland was conducted using the air quality database maintained by the Chief Inspectorate for Environmental Protection in Poland. A higher concentration of PM_2.5_ was associated with a decrease in birth weight and a higher risk of low birthweight (i.e., <2500 g). We also observed lower APGAR scores. Thus, all possible efforts to reduce air pollution are critically needed.

## 1. Introduction

In 2019, the World Health Organization (WHO) placed both air pollution and climate change among the top ten global health hazards. Simultaneously, air pollution was pointed by the WHO as the single greatest environmental risk to health [1]. Each year, 7,000,000 people die prematurely from diseases that may be linked to indoor and outdoor air pollution [1].

Polish cities are among the most heavily polluted in Europe. According to the WHO Ambient Air Pollution Database, 33 out of the 50 most polluted cities in Europe are located in Poland [2]. Consequently, 43,100 people are estimated to die prematurely due to air pollution in Poland every year [3]. However, the number may be much higher when ramifications of air pollution for future generations’ life and health are considered. The developing fetus is highly susceptible to the hazardous chemical compounds contained in the air inhaled by a pregnant woman. Toxic compounds contained in the particulate matter of less than 2.5 μm in diameter (PM_2.5_) are transferred through the pulmonary alveoli to the blood flow of the exposed woman and, together with blood, reach placenta and fetal circulation, emitting a toxic influence to the fetal cells [4]. The developing fetus is highly susceptible to toxins, particularly during specific windows of gestation. The fetus is more susceptible than adults to the negative effects of the toxic and carcinogenic compounds due to its high absorption and retention of the aforementioned compounds and the difficulties in their metabolism and detoxification caused by the immature liver and immunological system. Furthermore, inefficient are also the DNA repair mechanisms of the fetus with simultaneous intensive cell proliferation at this stage of life. Moreover, these compounds may interfere with placenta and disrupt its transport mechanisms [5], which may cause the intrauterine growth restriction, preterm birth, birth defects, as well as DNA changes, leading to mutations and epigenetic changes [6]. This type of influence on the genetic material is multiplied during subsequent cell divisions, increasing the risk of chronic diseases and tumors in the extrauterine life [5].

In the global perspective, 25% of the PM_2.5_ air pollution is caused by fuel combustion. Another 15% is contributed to by industry—especially coal-based energy production. About 20% of the aforementioned fine particulate matter in the global air pollution is generated by households and the burning of coal, wood and various house waste. The same percentage comes from unspecified sources of human origins and another 18% from natural dust and salt [7]. However, considering Poland, it is believed that even 50% of the PM_2.5_ air pollution is attributed to the household sector [8]. Consequently, the cold months of the year are a period of significantly increased concentration of the fine particulate matter in the air—especially on windless days. One such situation was observed in Poland at the turn of the years 2016 and 2017, when the concentration of PM_2.5_ reached levels comparable to Beijing and New Delhi [2].

The objective of this study is to evaluate the effects of the particulate matter air pollution of less than 2.5 μm in diameter on the pregnancy outcomes.

## 2. Materials and Methods

An analysis of pregnant women and their children was conducted using survey questionnaires from the Polish pregnancy-related assessment monitoring system (Pol-PrAMS) program. This population-based study was conducted between 2 February and 22 March 2017 in all hospitals in Poland. Groups of Polish women and their newborns were surveyed in all of the hospitals in Poland during postpartum hospitalization. Thus, all of the women hospitalized postpartum on the designated days of the study were deemed eligible for the study. The ethics committee of the Institute of Rural Health in Lublin, Poland, approved the study (reference number 03/2011). Participation was anonymous and voluntary. The survey was divided into two parts. The first contained over 70 questions, namely, the age, place of residence, education, social and economic status and health behaviors before and during pregnancy. Mothers who stayed in hospitals after birth completed the first part of the survey. The second part had nine questions that were completed by the medical personnel providing healthcare to the mother and newborn, with the use of medical records (pregnancy cards and patient medical history). The questions in this part concerned the childbirth method, newborn status after birth and birth defects. It also included questions about the results of laboratory tests performed on mothers and newborns after birth. The methodology of this study is described in detail in a separate articles [9,10].

Overall, 3451 women who gave birth and their newborns were hospitalized in obstetric wards of the surveyed hospitals in Poland. Our sample consisted of women who gave birth in cities where the fine particulate matter monitoring stations were located. Additionally, only term pregnancies were included, i.e., those who ended between the full 37th and the incomplete 42nd week of gestation. Thus, our sample consisted of 1095 women.

An analysis of air pollution in Poland in the period from 15 April 2016 to 22 March 2017 was made using the air quality database maintained by the Chief Inspectorate for Environmental Protection in Poland. The database contains records of the average daily concentration of PM_2.5_ throughout the entire year in the cities where monitoring stations are located. There are 64 stations of this type in Poland, but we included in the analysis 50 cities in which women gave birth during the study period. For each city, the average concentration of PM_2.5_, for the period between the 15 April 2016 and the 22 March 2017, was calculated. The reason for selecting this period was the fact that all of the women in our sample were pregnant at that time. It was assumed that women who were admitted to the hospital at the time of the survey on the 2 February 2017 and who gave birth before the 42nd week of pregnancy, had their last period on the 15 April 2016, as per Naegele’s rule. The last woman applicable for the survey would most likely give birth on the 22 March 2017, on the last day of the Pol-PrAMS study. Women taking part in the survey were divided into two groups. The first consisted of those who gave birth in cities where the average concentration of PM_2.5_ was no higher than 25 µg/m^3^, which is the acceptable average level per annum, as indicated in the directive of the European Union [11]. The other group consisted of women who gave birth in cities polluted with the average fine particulate matter concentration higher than 25 µg/m^3^.

Continuous variables were compared using Mann–Whitney–Wilcoxon Test, while a chi-squared test was applied for categorical variables. The results were expressed as the mean and standard deviation and 95% confidence intervals (95% CI) or as a frequency (%). Logistic regression models were created to estimate the odds ratios and 95% CI. For each analyzed outcome, the crude odds ratio was computed for the single predictor: index of exposure to fine particulate matter (PM_2.5_) concentration higher than 25 µg/m^3^. In multivariable models the effect of exposure was adjusted to the effects of education, economic status, social conditions, cigarette smoking, alcohol consumption and presented as adjusted odds ratio (aOR). All statistical analyses were performed using IBM SPSS software version 25 (IBM Corp., Armonk, NY, USA). A *p*-value of <0.05 was considered statistically significant.

Each time we discuss gestational diabetes mellitus (GDM) in the results of our work, it refers to diabetes diagnosed for the first time during pregnancy in patients who did not suffer from this disease before pregnancy [12]. At the same time, we only consider pregnancy-induced hypertension (PIH), i.e., hypertension that was diagnosed for the first time after the 20th week of the current pregnancy [13].

## 3. Results

Supplementary Figure S1 presents the concentration of PM_2.5_ in the period between the 15 April 2016 and the 22 March 2017 in the analyzed cities in Poland.

Approximately 1 in 3 women was between 31 and 35 years old (34.4%). Almost the same percentage referred to women aged between 26 and 30 years old (34.1%) The highest percentage corresponded to women with a higher education degree (62%), over 33% were women who had completed secondary education, and almost 5% had finished primary school education. More than half of the women considered their financial situation to be good. Over 40% of women described their social conditions to be very good or good (respectively 40.7% and 49.9%). Almost 69% of the women declared to have never smoked, the second largest group were women who had stopped smoking for the duration of pregnancy (14.5%). Simultaneously, 5.1% reported that they have been smoking during pregnancy. Slightly more than 40% of women admitted to consuming a certain amount of alcohol during pregnancy. The pregnancy of 7.1% had complications linked to GDM and 5.7% had high blood pressure-related complications. Both analyzed groups differed in regard to education, financial conditions and social conditions. Patient characteristics are shown in Table 1.

The average weight and birth length of newborns that were born in the areas of the average fine particulate matter pollution ≤25 µg/m^3^ were 3478 g and 55.0 cm, respectively. In newborns born in areas where the concentration of the fine particulate matter was >25 µg/m^3^, the birthweight was lower by 115 g (*p* < 0.05) and the length of the infant was 0.4 cm shorter (*p* > 0.05). The aforementioned details are shown in Table 2.

Heightened PM_2.5_ concentration was associated with an increase in the number of births in which the newborn weight was lower than 2500 g (*p* < 0.05). Infants born in cities where fine particulate matter pollution was >25 µg/m^3^ had Apgar scores of less than 8 more frequently, tested between 1 and 10 min after birth (*p* < 0.05). However, in this group there was an increase in the percentage of women whose pregnancy or labor was complicated by one the following pathologies: birthweight lower than 2500 g, premature rupture of membranes (PROM), the Apgar scores of less than 8 (tested between 1 and 10 min after the birth) or diagnosed birth defects (<0.05). Nevertheless, no statistically significant effect of increased PM_2.5_ concentration was observed in relation to the following aspects: premature rupture of membranes, type of labor, birth defects, sex of the child, hospitalization during pregnancy (*p* > 0.05). Table 3 presents the unadjusted data on influence of air pollution on the obstetrical outcomes.

Results from models adjusted for maternal characteristics are reported in Table 4. These present the risk factors of the occurrence of different pregnancy outcomes. Children born in the areas of heightened air pollution were 4 times more likely to have a birthweight lower than 2500 g (OR = 4.3, 95%; CI: 1.5–12.3; *p* < 0.05) and almost twice more frequently scored less than 8 points in the Apgar score (OR = 2.4, 95% CI: 1.2–4.6 *p* < 0.05). In this group the following pathologies appeared more often: low birthweight or PROM or APGAR < 8 or birth defects (OR = 1.4, 95% CI: 1.1–1.9; *p* < 0.05). Results of tests of difference in PROM and cesarean section between areas with lower and higher levels of air pollution were not statistically significant. Surprisingly, a lower risk of the GDM development was observed (OR = 0.5, 95% CI: 0.3–0.9; *p* < 0.05).

## 4. Discussion

Air pollution with fine particulate matter of less than 2.5 µm in diameter has become a global health challenge. Over recent years, the number of studies analyzing the link between prenatal risks and further development of the fetus and child has increased.

Increased concentration of PM_2.5_ during the entirety of the pregnancy has a negative impact on birthweight [14]. This finding is consistent with results reported elsewhere [15,16]. Attention is also paid to the impact of air pollution during specific periods of pregnancy on lower birthweight. In his analysis of 400,000 children, Kurman observed a stronger negative impact of higher air pollution on the birth weight of children in the first trimester of pregnancy than in the remaining two trimesters [17]. Higher risk of a low birth weight (i.e., <2500 g) was also observed [18]. Details of this process are not yet fully understood. It may be due to the increased alveolar ventilation, which is a physiological phenomenon during pregnancy, but may cause increased absorption of air pollutants [19].

Research carried out by Maciel-Rutz et al. does not indicate any significant impact of PM_2.5_ on the Apgar scores in newborns [20]. In our study, the Apgar score of less than 8 was observed in newborns born in the cities more heavily polluted than the average 25 µg/m^3^. Nevertheless, it needs to be emphasized that the evaluation of the direct correlation between air pollution and the Apgar score is difficult to unambiguously conclude. The conditions of newborns are affected by numerous factors, like the course of birth or the accompanying illnesses of the mother or child.

Different conclusions were drawn from the analysis of the influence of air pollution on birth defects. There is a plentitude of research carried out with the goal of finding the link between these two factors. However, the results are inconclusive and require a further in-depth analysis in order to understand the mechanism of this phenomenon. It seems that the influence of air pollution is greatest in the critical periods of formation of particular internal organs during embryogenesis that is in the first trimester. Thus, our use of average ambient levels of PM_2.5_ over the previous year may not have captured meaningful variations in pollutant levels during periods of critical fetal development relevant for these outcomes

Vrijheid, M. et al. proposed a hypothesis that inhalation of pollution and its particles leads to their absorption into the blood flow, which causes the oxidative stress that damages the cells’ DNA. Furthermore, the inhaled compounds may contribute to a local inflammatory response in placenta, cause changes in the coagulation system, as well as influence the processes of migration and differentiation of neural crest cells [21].

There are many research papers describing the effects of air pollution on the structure and performance of the hearth. Tanwar et al. and Hall et al. pointed out that the exposure to PM_2.5_ during the prenatal period causes significant disruptions of the heart structure and activity in adulthood. The risk of these complications is especially high when the exposure takes place between the 2nd and the 8th week of pregnancy, during the period of development of the cardiovascular system [22,23]. Other research indicates an increased risk of pulmonary stenosis or ventricular septal defect [24] as well as coarctation of the aorta [25]. Other observations include a heightened risk of defects in the digestive system [25], formation cleft palate [26] or an increased risk of genital defects [27]. Nevertheless, our study does not conclude the existence of the link between air pollution and heightened risk of birth defects in a child.

Many studies indicate a link between air pollution and increased risk of developing gestational diabetes. The risk is connected to the increased concentration of NO during the first and the second trimester of the pregnancy [28], SO_2_ during the first trimester (particularly between the 4th and the 10th pregnancy week) [29] and PM_2.5_ in the second trimester [30]. Our results are inconsistent with previously reported findings, as this research revealed a lowered risk of gestational diabetes mellitus in women living in cities with a high PM_2.5_ concentration. The reason may be the methodological differences of abovementioned studies. Two of the three previous studies cited, used different pollutants (NO and SO_2_) and all used different time periods for indicators of exposure (trimester versus year-long period). It seems likely that these differences could contribute to differences in patterns of results.

The limitations of this study include the estimation of PM_2.5_ exposure in pregnant women. According to the methodology, the risk stems from the average concentration of PM_2.5_ in the place of delivery. This does not necessarily mean that the expectant woman stayed within this place for the entire pregnancy. The women were not asked for how long they had stayed in a city where a monitoring station was placed. Thus, our results are only an estimate of women’s exposure to air pollution. Moreover, the air pollution we took into account is the mean air pollution from the entire period under study, i.e., between the 15 April 2016 and the 22 March 2017. Additionally, it needs to be remembered that both of the samples were not identical. We tried to eliminate the differences in the structures of both groups by taking into account such variables as education, economic status, social conditions, smoking and alcohol consumption in the logistic regression models. Despite these limitations, this is the first and only research of this type in Poland that analyses the effects of air pollution on pregnancy outcomes in such a large sample.

## 5. Conclusions

Our study adds to a larger body of evidence showing adverse effects of air pollution for birth outcomes. Air pollution may impact pregnancy outcomes in terms of lower birth weight, higher risk of giving birth to a child weighing <2500 g and also to a child in a worse general condition defined as APGAR scores. Yet, this study limitations do not allow us to draw indisputable conclusions. Further study is warranted to address some inconsistencies in findings across studies and gaps in the literature, but that based on the body of evidence a failure to address excess exposure to PM_2.5_ contributes to poor birth outcomes among children exposed in utero. These poor birth outcomes have been associated with heightened medical risk throughout the life course [30,31]. Thus, efforts to reduce air pollution are likely to not only promote better birth outcomes, but also contribute to improved health over the life course.

## Figures and Tables

**Table 1:** Patient characteristics.

		Average Annual PM_2.5_ Concentration (µg/m^3^)						
		≤25 µg/m^3^		>25 µg/m^3^		All		*p*
		N	%	N	%	N	%	
**Age**								ns
<25		104	16.1%	71	16.2%	175	16.2%	
26–30		222	34.4%	147	33.6%	369	34.1%	
31–35		228	35.4%	144	33.0%	372	34.4%	
>35		91	14.1%	75	17.2%	166	15.3%	
**Education**								<0.05
Basic		32	5.1%	19	4.4%	51	4.8%	
Secondary		188	29.8%	164	38.2%	352	33.2%	
Higher		410	65.1%	246	57.4%	656	62.0%	
**Economic Status**								<0.05
Very good		156	24.1%	77	17.4%	233	21.4%	
Good		372	57.5%	287	64.8%	659	60.4%	
Average/bad		119	18.4%	79	17.8%	198	18.2%	
**Social Conditions**								<0.05
Very good		289	44.7%	154	34.8%	443	40.7%	
Good		298	46.2%	245	55.5%	543	49.9%	
Average/bad		59	9.1%	43	9.7%	102	9.4%	
**Cigarette Smoking**								ns
Yes		39	6.1%	16	3.7%	55	5.1%	
Quit during pregnancy		99	15.5%	56	13.0%	155	14.5%	
No, from several years		71	11.1%	52	12.0%	123	11.5%	
No, never		429	67.3%	308	71.3%	737	68.9%	
**Alcohol Consumption**								ns
Never		374	57.5%	265	59.6%	639	58.3%	
At most once per month		169	26.0%	99	22.2%	268	24.5%	
Twice per month and more often		107	16.5%	81	18.2%	188	17.2%	
**Gestational Diabetes Mellitus**								ns
No		598	92.0%	419	94.2%	1017	82.9%	
Yes		52	8.0%	26	5.8%	78	7.1%	
**Pregnancy-induced Hypertension**								ns
No		571	94.2%	396	94.5%	967	94.3%	
Yes		35	5.8%	23	5.5%	58	5.7%	

*p*-value refers to two groups of women with analyzed PM_2.5_ concentration; ns—statistically insignificant, *p* < 0.05.

**Table 2:** Birth weight and newborn length depending on PM_2.5_ concentration.

Variable	N	Mean	Mean Difference	S.D.	95% CI Lower Limit	95% CI Upper Limit	*p*-Value
**Weight (g)**							<0.05
PM_2.5_ ≤ 25 µg/m^3^	634	3478	0	446	3444	3513	
PM_2.5_ > 25 µg/m^3^	432	3363	−115	490	3317	3409	
**Length (cm)**							ns
PM_2.5_ ≤ 25 µg/m^3^	608	55.0	0	2.7	54.7	55.1	
PM_2.5_ > 25 µg/m^3^	418	55.0	−0.4	3.1	54.3	54.9	

Ns—statistically insignificant, *p* < 0.05.

**Table 3:** Pregnancy outcomes depending on average annual PM_2.5_ concentration.

	Average Annual PM_2.5_ Concentration (µg/m^3^)				*p*
	≤25 µg/m^3^		>25 µg/m^3^		
	N	%	N	%	
**Pregnancy Outcome**					
**Birthweight (g)**					<0.05
>2500	629	99.2	419	97.0	
<2500	5	0.8	13	3.0	
**PROM**					ns
No	454	71.6	282	66.4	
Yes	180	28.4	143	33.6	
**Type of Labor**					ns
Vaginal	387	59.8	250	57.1	
Cesarean section	260	40.2	188	42.9	
**APGAR**					<0.05
≥8	590	97.0	415	94.3	
<8	18	3.0	25	5.7	
**Birth Defects**					ns
No	520	98.7	363	99.5	
Yes	7	1.3	2	0.5	
**Sex of the Child**					ns
Female	306	47.9	202	47.8	
Male	333	52.1	221	52.2	
**Hospitalization**					ns
No	372	62.0	257	62.8	
Yes	228	38.0	152	37.2	
**Pathology**					<0.05
No	451	69.5	274	61.7	
Yes	198	30.5	170	38.3	

Legend: PROM—premature rupture of membranes; Pathology—low birthweight or PROM or APGAR score < 8 or birth defects; ns—statistically insignificant (*p* < 0.05).

**Table 4:** Adjusted odds ratios and 95% confidence intervals for different pregnancy outcomes among women exposed to fine particulate matter (PM_2.5_) concentration greater than 25 µg/m^3^.

	Adjusted Odds Ratio				
	aOR		95% CI		*p*
**Birthweight (g)**					<0.05
>2500	Reference				
<2500	4.3		1.5–12.3		
**PROM**					ns
No	Reference				
Yes	1.2		0.9–1.6		
**Type of Labor**					ns
Vaginal	Reference				
Cesarean section	1.2		0.9–1.5		
**APGAR**					
≥8	Reference				<0.05
<8	2.4		1.2–4.6		
**Birth Defects**					
No	Reference				ns
Yes	0.4		0.1–1.9		
**GDM**					<0.05
No	Reference				
Yes	0.5		0.3–0.9		
**PIH**					ns
No	Reference				
Yes	1.0		0.6–1.7		
**Hospitalization**					ns
No	Reference				
Yes	0.9		0.7–1.2		
**Pathology**					<0.05
No	Reference				
Yes	1.4		1.1–1.9		

Legend: PROM—premature rupture of membranes; GDM—gestational diabetes mellitus; PIH—pregnancy induced hypertension; PPH—pregnancy hypertension; pathology—low birthweight or PROM or APGAR < 8 or birth defects; ns—statistically insignificant (*p* < 0.05).

## Supplementary Materials

The following are available online at https://www.mdpi.com/1660-4601/17/16/5820/s1, Figure S1: Concentration of PM_2.5_ in analyzed cities in Poland, between 15 April 2016 and 22 March 2017.
