# Supplementary material for: Prenatal Fine Particulate Matter (PM2.5) Exposure and Pregnancy Outcomes—Analysis of Term Pregnancies in Poland
Source: Int J Environ Res Public Health. 2020 Aug 11;17(16):5820. doi: 10.3390/ijerph17165820 (PMC7459454; doi:10.3390/ijerph17165820)
Supplement: Supplementary file 1 [file ijerph-17-05820-s001.pdf]

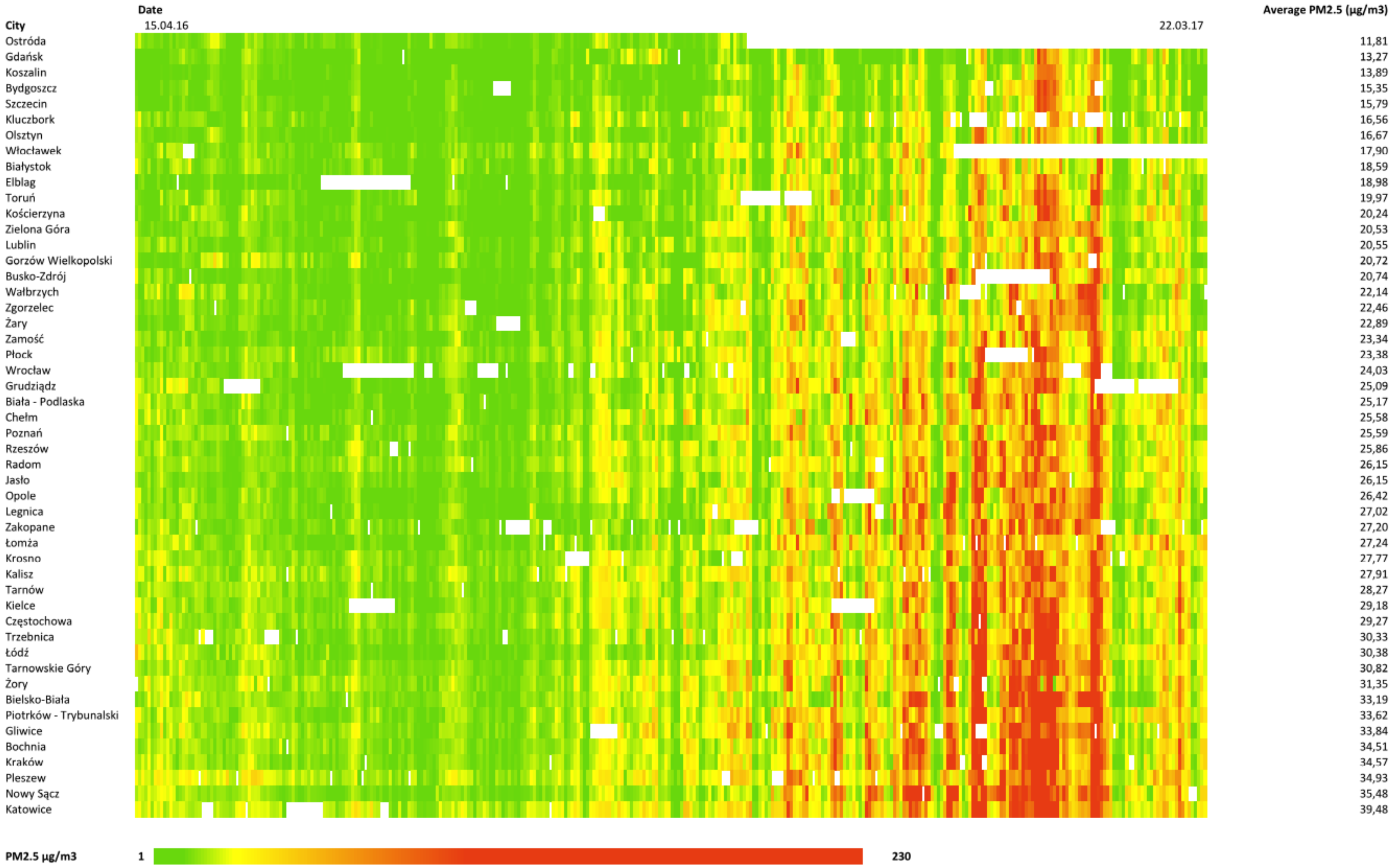

Figure 1. Concentration of PM2.5 in analyzed cities in Poland, between 15th of April 2016 and 22nd of March 2017.
